# Hypothesis: Immunotherapy by Selective Convalescent Blood Engineering to Stifle Diseases like COVID-19

**DOI:** 10.7150/ijms.46363

**Published:** 2021-08-01

**Authors:** Arka Prava Mukherjee

**Affiliations:** Greka Engineering and Technology, 28 Landmark Plaza, Outer Ring Road 1, CBD, Zhengzhou, 450000, China.

**Keywords:** Convalescent Blood, WBC, Leukapheresis, Transfusion therapy, Vaccination, COVID-19

## Abstract

Current standard vaccine testing protocols take approximately 10-24 months of testing before a vaccine can be declared successful. Sometimes by the time a successful vaccine is out for public use, the outbreak may already be over. With no vaccine or antiviral drug available to treat the infected, we are left with the age-old methods of isolation, quarantine, and rest, to arrest such a viral outbreak. Convalescent blood therapy and covalent plasma therapy have often proved effective in reducing mortality, however, the role of innate and adaptive immune cells in these therapies have been overlooked. Antigen presenting cells (APCs), CD4+ T memory cells, CD8+ T memory cells, and memory B-Cells all play a vital role in sustainable defense and subsequent recovery. This report incorporates all these aspects by suggesting a novel treatment therapy called selective convalescent leukapheresis and transfusion (SCLT) and also highlights its potential in vaccination. The anticipated advantages of the proposed technique outweigh the cost, time, and efficiency of other available transfusion and vaccination processes. It is envisioned that in the future this new approach could serve as a rapid emergency response to subdue a pathogen outbreak and to stop it from becoming an epidemic, or pandemic.

## Introduction

Four factors can make a novel pathogen outbreak extremely deadly: a) high reproduction number or Ro factor (Ro≥3); b) high mortality rate (≥50%); c) long incubation period (≥10 days); and d) asymptotic spreading. If a pathogen has any three of these four characteristics, it can be classified as Pathogen-X, i.e., a pathogen which has the potential to cause mass extinction. One of the hypotheses to explain past mass extinctions of certain species (recent and geological) is highly infectious disease outbreak 1-5.

Currently the world is facing one of the worst pandemics in human history. As of 25^th^ July 2021 the officially reported cases of Corona Virus Pandemic or COVID-19 has touched nearly 194 million with about 4 million related deaths worldwide. Not to mention the billions of lives who are suffering due to the adverse effects of economic turmoil and disruption of normal livelihood; and there seems to be no end in sight. As scientists we can ask ourselves: Shouldn't we be better prepared? Isn't this a wakeup call for the scientific community?

Current standard vaccine testing protocols take nearly 10-24 months of testing before a vaccine can be declared successful. Sometimes by the time a successful vaccine is available for public use, the outbreak may already be over. A good example is the 'Spanish flu' or the 1918 influenza pandemic, which wreaked havoc over a short span of two years. The flu was caused by an H1N1 virus with genes of avian origin and can be categorized as the most severe pandemic in the last two centuries. Spanish flu infected around 500 million people worldwide, or one-third of the world's population, and nearly 50 million people died 6. No vaccine or antiviral drug was available to treat the infected. Isolation, quarantine, and rest were applied to arrest the viral outbreak. However, doctors around the world also used blood products from survivors, which helped to reduce the mortality rate by as much as 50%. Similar approaches using 'convalescent blood plasma therapy' have been reported in the treatment of viral outbreaks like Ebola 7,8 (Mupapa et al., 1999; van Griensven et al., 2016).

Can the human race (species) afford long testing protocols when struck with a deadly pathogen like a novel pathogen-X? This paper suggests a hypothesis of a possible option to minimize the rate the spread of a disease like COVID-19, if not completely stop it.

## Hypothesis

**Figure 1:**
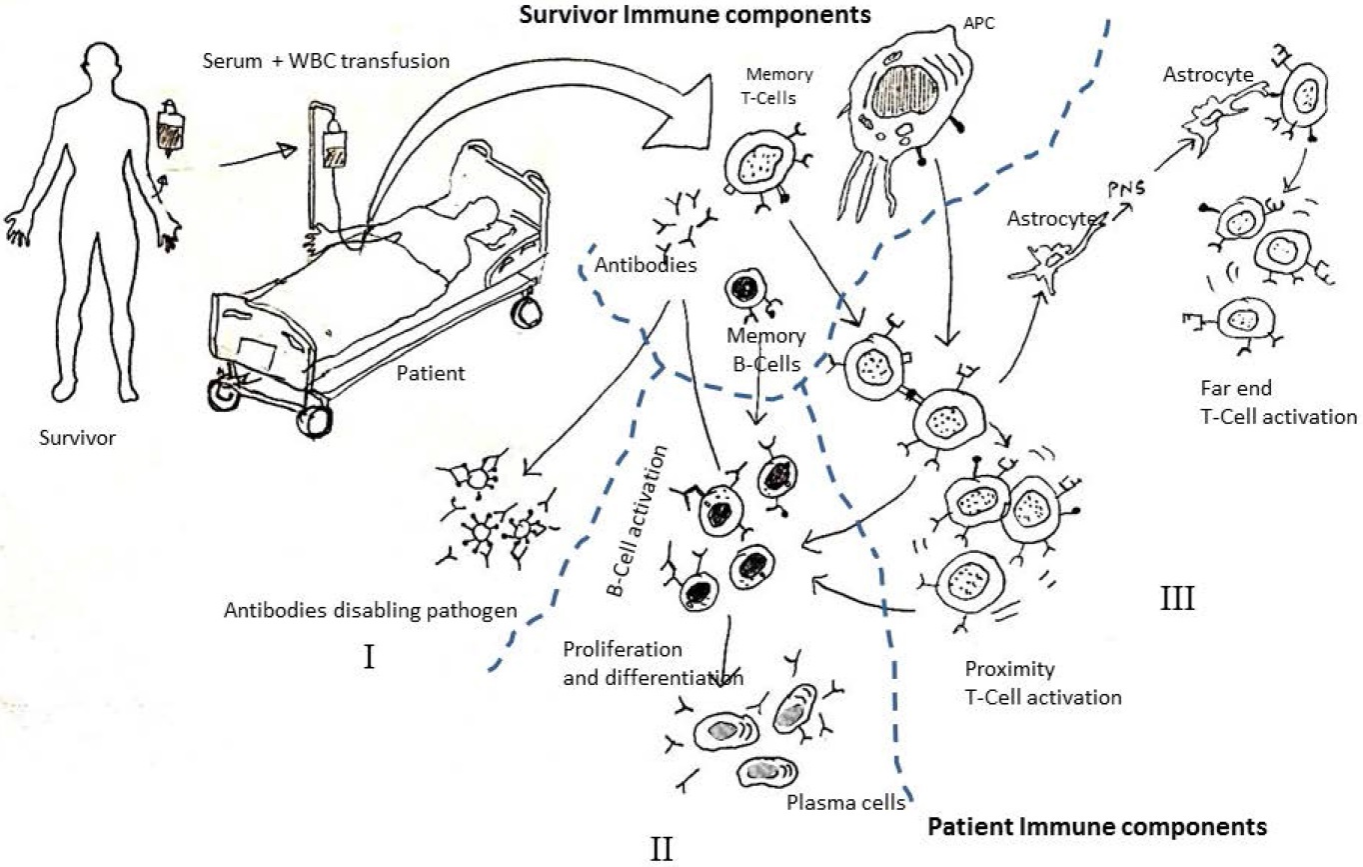
Hypothesis on what might be happening during convalescent blood transfusion therapy. Reprinted from Mukherjee 9.

Techniques like 'monoclonal antibody technique' or 'covalent plasma therapy' all focuses on administration of 'antibodies' from survivors to patients. However, during these transfusion techniques, little attention is paid to the generating mechanism of the antibodies. When convalescent blood transfusion therapy is applied, one, or all, of the following three things could be happening (Figure 1):

- **Reinforcement**: by injecting 500 mL or more of the survivor's blood the patient's immune system is reinforced by increasing the number of immune cells and antibodies;
- **Knowledge transfer**: information encoded in the white blood corpuscles (WBC) of the survivor's immune (**SImmune**) system, such as antigen presenting cells (APCs), memory CD4+ T Cells , CD8+ T cells, and memory B cells, quickly trigger a response in the patient's immune (**PImmune**) system (innate and/or adaptive). For example, antigen (like Spike protein) expression on the convalescent blood APCs can trigger a very quick response from the T-cells of PImmune system, creating a sustainable defense mechanism and reducing the turnaround time of effective response 9.
- **Cytokine transfer:** cytokines already present in the survivor's blood quickly trigger an immune response when injected into a patient to produce the right kind of antibodies required to fight off the pathogen.

Some medical scientists argue that factor I is the only factor responsible for a successful recovery. It is unclear whether 500 mL or 1 L of new blood holds sufficient antibodies to overrun the pathogen population without a further increase in their numbers. Antibodies cannot generate more antibodies, unless there is a copying mechanism that is still unknown to medical science. Therefore, it is seems most likely that 'knowledge transfer' (factor II) leads to a sustainable defense (Figures 1 & 3) and subsequent recovery. Some recent research reviews in medical science 16,17 (viz. Grifoni et. al 2020, Sette and Crotty 2020) seem to support this conjecture (factor II) and highlights the possible role memory CD4+ and CD8+ T-cells in the immune response against COVID-19. Furthermore, studies of techniques such as T-cell engineering 12 highlight the importance of factor II in therapeutic uses against cancer cells.

### Proposed new approach: Selective Convalescent Leukapheresis and Transfusion (SCLT)

Based on the aforementioned hypothesis, I propose selective convalescent leukapheresis and transfusion (SCLT) as a rapidly deployable therapeutic treatment for highly infectious diseases like COVID-19, Ebola, influenza, SARS, and MERS. The technique would involve:

*Stage 1:* selection of a suitable donor, i.e., a survivor with a strong immune system. In other words convalescent blood should be taken from survivors who exhibited mild symptoms, quick recovery, and have no underlying conditions or other transmittable disease like HIV, HPV, etc.

**Figure 2:**
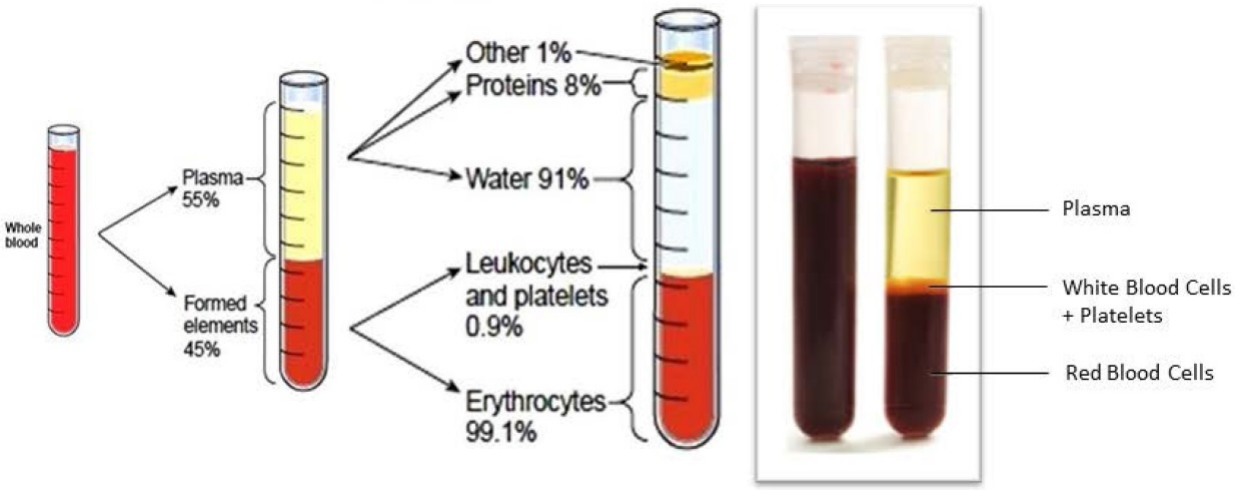
Whole blood and its components. Courtesy: http://givingblood.org/about-blood/blood-components.aspx.

**Figure 3:**
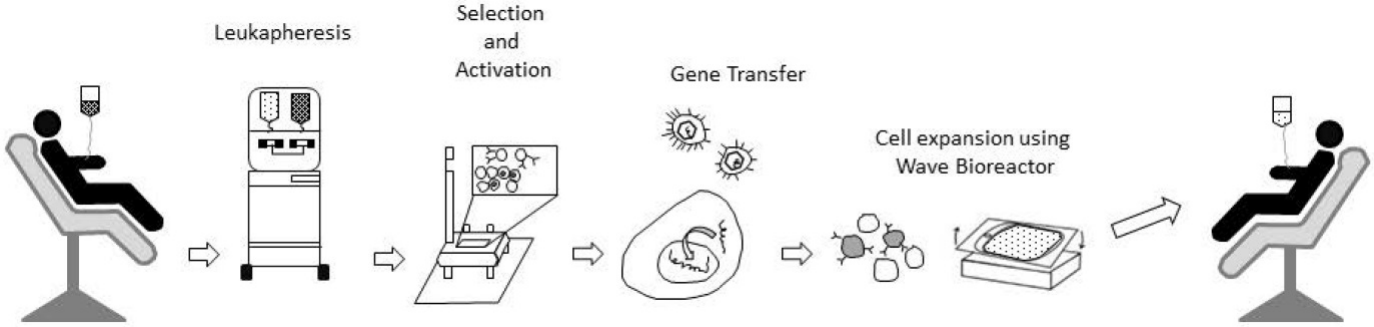
Schematic steps for Leukapheresis and enrichment process in engineered T-Cell therapy for cancer treatment. For details please refer to research papers like Fesnak et al. 12.

*Stage 2:* the whole blood (Figure 2) should be subjected to fractional separation by suitable leukapheresis processes 12 to extract the convalescent WBCs (Figure 3). If required, this process could be repeated by collecting convalescent blood from different donors until sufficient convalescent WBC was available for transfusion.

*Stage 3:* the transfusion of the convalescent WBC. The convalescent WBC can be administered in different quantities based on whether it is used for therapeutic treatment or for the purpose of vaccination (Table 1).

The proposed SCLT technique has the following potential advantages:

- **Vaccination:** It is important to note that this treatment pathway can also be used as a novel vaccination process. APCs present in the convalescent WBC can trigger an immediate immune response in a healthy host, resulting in the creation of millions of antibodies. For vaccination purposes, only a small quantity of enriched convalescent WBC would be needed (Table 1);
- **Sustainable defense:** this technique helps the patient's immune system by providing the information required for a sustainable fight against a pathogen attack;
- **Low risk of natural attenuation:** while antibodies present in the convalescent blood plasma have the risk of natural attenuation over time (as observed in some discouraging results of convalescent blood plasma transfusion 13), the WBC components in the Convalescent Blood, like CD4+ and CD8+ memory cells and APCs, have a much longer natural life and provide much needed protection from future attacks from the same pathogen. Thus the chances of success in the SCLT are much more than just convalescent blood plasma transfusion.

- **Time efficiency:** the technique reduces the turnaround time of effective response in the PImmune system by the process of quick 'knowledge transfer' (as mentioned in factor II). Even when compared to recent mRNA vaccination processes, this treatment/vaccination pathway would be much faster as the APCs in the convalescent WBCs are ready with pathogen epitope expressions (like the COVID-19 Spike protein).
- **Quantity efficiency:** a smaller quantity of *in vitro* injection would be required in SCLT as compared with 'convalescent whole blood' or 'convalescent blood plasma' transfusion techniques.
- **Reduced time for FDA approval:** even when compared to recent mRNA vaccination processes, the SCLT treatment/vaccination pathway should face fewer hurdles in getting FDA approval as it is ethically similar to the 'convalescent whole blood' transfusion technique and 'Convalescent Blood plasma' transfusion techniques.
- **Lower cost:** this technique would be lower cost as it involves simple fractionation, enrichment, and administration processes.

Clearly, the possible side effects of GVD must be carefully considered prior to administration of WBC for therapeutic use. Furthermore it is very important to note that the SCLT technique in not a panacea and is just an alternative approach which can be used for vaccination and treatment against diseases like COVID-19.

### Recent Developments and Testing of SCLT technique

Since the release of papers Mukherjee 2020 10, 11 in March 2020 in open access journals; the SCLT hypothesis has been scrutinized by experts; and its EQA (External Quality Assessment) is currently being tested in some medical laboratories/organizations like Saint Louis University Center for Vaccine Development and Duke Health (Duke University Health System). Call for human trials 14, 15 have already begun for vaccine development.

## Conclusion

In the absence of readily available vaccines or cures against a deadly novel pathogen, SCLT could not only act as a first line of defense but could potentially be used to vaccinate human populations against diseases like COVID-19, influenza, SARS, MERS, Ebola, etc. If applied correctly, this technique may be able to prevent a pathogen disease outbreak from turning into an epidemic or pandemic. However, before trying this technique on humans, it is mandatory that medical scientists must carefully consider the side effects of WBC related transfusion.

## Figures and Tables

**Figure 4:**
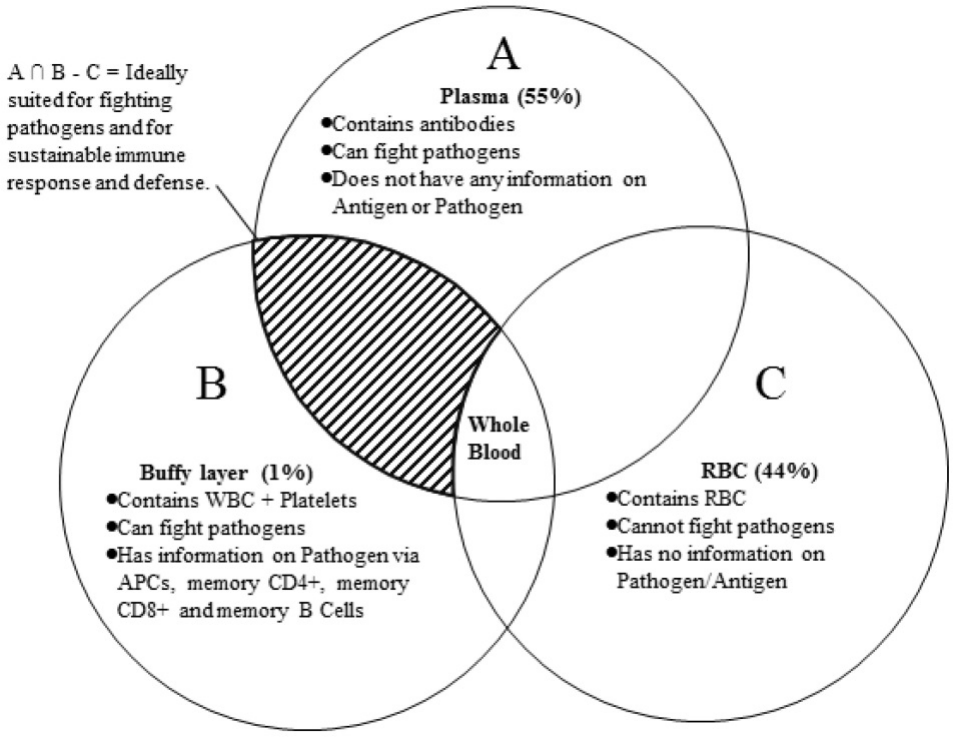
Blood components and simple Boolean logic to emphasize the importance of convalescent white blood cells (WBCs).

**Table 1:** Anticipated dosage of white blood cell (WBC) + plasma from convalescent blood (caution: must be tested clinically for safe use)

	Patient age group 0-10 years	Patient age group 11-50 years	Patient age group 51-65 years	Age >65 years
Already infected patient (for treatment)	Dosage: small amt. of WBC + small amt. of Plasma	Dosage : medium amt. of WBC + medium amt. of Plasma	Dosage: medium amt. WBC + large amt. of Plasma	Dosage: medium amt. WBC + very large amt. of Plasma
	Critical Condition: 2 dosage/day	Critical Condition: 3 dosage per day	Critical Condition: 4 dosage per day	Critical Condition: 4 dosage per day
	Normal Condition: 1 dosage/day	Normal Condition: 2 dosage per day	Normal Condition: 3 dosage per day	Normal Condition: 3 dosage per day
	Mild Condition: 1 dosage/day	Mild Condition: 1 dosage per day	Mild Condition: 2 dosage per day	Mild Condition: 2 dosage per day
Healthy Person (for vaccination)	small amt. of WBC	small amt. of WBC	small amt. of WBC	small amt. of WBC

Here, WBC: WBC derived from convalescent blood.
